# Exploring Alternative Splicing in Response to Salinity: A Tissue-Level Comparative Analysis Using *Arabidopsis thaliana* Public Transcriptomic Data

**DOI:** 10.3390/plants14071064

**Published:** 2025-03-30

**Authors:** Jesús Hernández-Urrieta, José Miguel Álvarez, José Antonio O’Brien

**Affiliations:** 1 Facultad de Ciencias Biológicas, Pontificia Universidad Católica de Chile, Avenida Libertador Bernardo O’Higgins 340, Santiago 8331150, Chile; jmhernandez5@uc.cl; 2 Departamento de Fruticultura y Enología, Facultad de Agronomía y Sistemas Naturales, Pontificia Universidad Católica de Chile, Vicuña Mackenna 4860, Santiago 7820244, Chile; 3 Centro de Biotecnología Vegetal, Facultad de Ciencias de la Vida, Universidad Andrés Bello, Santiago 8370251, Chile; jose.alvarez.h@unab.cl; 4 ANID–Millennium Science Initiative–Millennium Institute for Integrative Biology (iBIO), Santiago 7500565, Chile; 5 ANID–Millenium Science Initiative Program–Millenium Nucleus in Data Science for Plant Resilience (Phytolearning), Santiago 8370186, Chile

**Keywords:** alternative splicing, salt stress, differential alternative splicing (DAS), tissue-specific regulation, abiotic stress

## Abstract

Increased soil salinity is a major threat to global agriculture and food security, caused mainly by anthropogenic activities and changing climatic cycles. Plants responses to salinity involve multiple regulatory layers, from transcriptome reprogramming to proteomic and metabolomic changes. Alternative splicing (AS) plays a role in coordinating the response to salinity, yet its extent, tissue, and condition specificity, remain poorly understood aspects. In this study, we used 52 publicly available RNA-seq datasets of salinity treatment to identify differential alternative splicing (DAS) events and genes participating in the response to this stimulus. Our findings reveal that either independently or coordinately, AS can regulate up to 20% of the transcriptome detected in *Arabidopsis*, with treatment intensity being the most determining factor. Moreover, we show that AS regulation was highly tissue-specific, with roots displaying strong AS-mediated stress responses. Furthermore, cross-stress comparisons showed that roots have a core set of AS-regulated genes associated with stress response and development, with functionally distinct sets of genes when comparing salt with other stresses, while also conserving a relevant condition-specific response. We demonstrate the need to integrate AS analysis to better understand plant adaptation mechanisms and highlight the key role of AS in salinity responses, revealing shared AS regulation between salt, heat, and drought responses.

## 1. Introduction

Increased soil salinity is a growing concern in agriculture nowadays, as it is one of the major factors contributing to the loss of productivity in cultivated land worldwide [1]. Salt stress affects plants mainly through water deficit (osmotic stress), ion imbalance (ionic stress), and oxidative stress [2]. However, it can also cause nutritional and metabolic alterations, trigger membrane and cell wall reorganization, and cause a reduction in cell division and expansion [3].

Salt sensing begins at the roots, which are first exposed to the stimuli in the soil. It involves the detection of four main signals: reduced soil water potential, high concentration of ions (mostly Na^+^ and chloride, Cl^−^), subsequent alteration of intracellular calcium (Ca^+2^) levels, and overall reactive oxygen species (ROS) accumulation (mostly H_2_O_2_ and O^−2^) [4,5,6]. Plants then respond through a series of morphophysiological changes that help to enhance tolerance and minimize damage [2,7,8]. Most response mechanisms studied in glycophytes are associated with ion transport (recovery of potassium, K^+^, homeostasis and exclusion or compartmentalization of Na^+^), ROS scavenging, and synthesis of osmolytes [2].

Underlying these response mechanisms, there are major transcriptional and post-transcriptional changes taking place during the response to salt stress [9]. Transcriptomic changes in this stress have been documented in the past either by genome-wide expression profiling using microarrays [10], or case by case through more conventional methodologies [11]. However, there are few studies showing the specific expression profile of *Arabidopsis* roots in response to salt stress using high throughput sequencing technologies. Furthermore, the post-transcriptional involvement in the salt stress response is understood to an even smaller extent, with emerging evidence pointing to a central role of alternative splicing (AS) in plant abiotic stress tolerance [12].

Precursor messenger RNA (pre-mRNA) splicing is a co-/post-transcriptional regulation process inherent to all eukaryotic organisms. It is carried on in the nucleus by a multidalton dynamic ribonucleoprotein complex called the spliceosome [13,14]. With the help of different splicing factors, the spliceosome assembles on specific canonical splice sites (SSs) within the pre-mRNA sequence, thereafter excising intronic regions and leaving only exons to be ligated into the mature mRNA [15]. Through this mechanism, constitutive splicing generates a defined transcript (usually the one with the strongest SSs). However, in AS, several different transcripts are rendered due to the recognition of alternative 3′ or 5′ SSs (or sets of both SSs) [12]. This alternative recognition has been shown to be influenced by environmental factors [16], and it results in at least four distinct AS types: intron retention (IR), exon skipping (ES), alternative five prime splice site (Alt5′SS), or alternative three prime splice site (Alt3′SS).

AS can, in part, explain the discrepancy between the number of proteins versus coding genes and has been regarded as a means of increasing proteomic diversity to deal with physiological, developmental, and environmental changes in plants [17]. By modifying mRNA sequences, AS can generate different protein isoforms that can have distinct functional properties. Additionally, depending on the type of sequence modification and where it occurs within the mRNA, AS can influence RNA stability, localization, and abundance, thereby contributing to gene regulation beyond protein diversity [13].

This regulatory mechanism has been proposed to be of importance in plant stress responses. During salt stress in *Arabidopsis thaliana*, there is a global increase at the whole seedling level in the pre-mRNA processing by AS [18], with up to 6000 genes changing their AS patterns [19]. Similar changes in AS events during salt stress have, since then, been observed in genome-wide studies in date palm [20], grapevine [21], cotton [22], rice [23,24], wheat [25], and barley [24]. AS regulation has also been reported under other abiotic stresses, such as high and low temperature stresses [26,27], and drought stress [28], suggesting a broader role in stress adaptation.

In the last decade, increasing evidence has shown that AS is not a passive consequence of stress but rather an actively regulated process that can be coordinated with signaling pathways. For example, splicing factors such as serine/arginine (SR) rich proteins and RNA-binding proteins (RBPs) are regulated at the transcriptional and splicing levels in response to abscisic acid (ABA) signaling and abiotic stress conditions [29]. This regulatory integration suggests that AS serves as a component of plant’s adaptive response to environmental stressors.

In salt stress and other abiotic stresses, one of the main challenges to functional characterization is the high variability in experimental conditions across studies. Factors such as plant developmental stage, environmental conditions, treatment parameters and tissue type have been shown to influence the transcriptome image, making it difficult to derive general conclusions about AS regulation in response to salinity from single-experiment approaches. To overcome this heterogeneity, integrating transcriptomic datasets from multiple studies can provide a more comprehensive and robust understanding of AS regulation in salinity responses [30]. In this study, we address this by systematically analyzing publicly available RNA-seq datasets to investigate the role of AS in salinity responses, assessing its extent, tissue specificity, cross-stress conservation, and experimental determinants.

## 2. Results

### 2.1. Summary of the Selected Datasets

Plant responses to increased salinity have been widely studied over the years. As a result, there is a large amount of Next Generation Sequencing (NGS) data generated upon applying salinity treatments to Arabidopsis plants. Leveraging the power of this publicly available information, we set out to inquire about the extent and form of alternative splicing regulation in response to salinity. For this, we performed a comprehensive search for all the available NGS datasets of different tissues and experimental salt treatment conditions. Thus, we were able to identify a total of 32 RNA-seq SRA BioProjects that used short-read sequencing from the year 2016 up to 2024. From these, 52 distinct comparisons were derived, encompassing 136 sequencing libraries. Samples came from four different plant tissues: roots, rosettes, whole seedlings, and seeds (Figure S1). Moreover, these BioProjects include experiments that were generated in a variety of experimental and biological conditions, from which salt treatment intensity, treatment duration, and plant age at treatment were mostly variable, as summarized along with other parameters in Table 1 and detailed in Table S1.

### 2.2. Alternative Splicing as a Regulatory Mechanism in Salinity Responses

To compare how AS responds to salinity, we analyzed the 32 selected studies and identified the differentially expressed genes (DEGs), differential alternative splicing (DAS) events, and the DAS genes. The latter considered genes whose transcripts undergo one or more AS events that significantly change between two experimental conditions, meaning they respond to the salt treatment.

**Figure 1:**
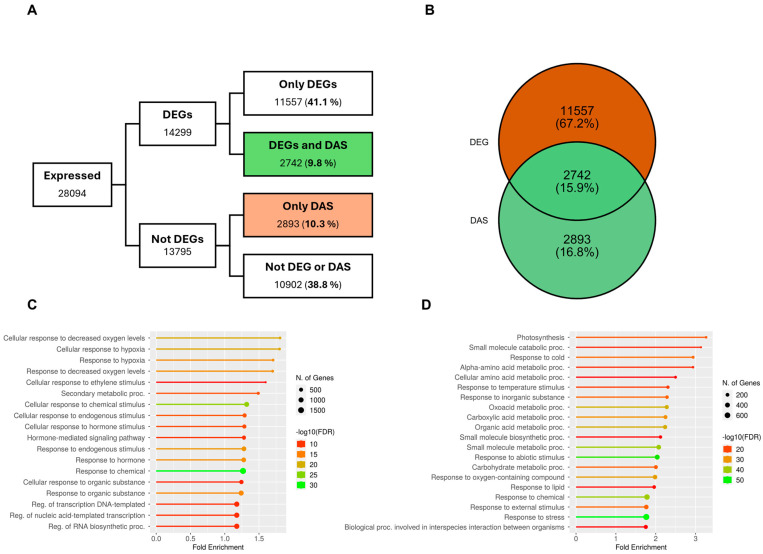
Comparison of alternative splicing and differentially expressed genes in response to salt treatments. (**A**) Sets of genes from the union of all datasets under different types of transcriptional regulations, DEGs: Differentially expressed genes, DAS: Differential alternative splicing. (**B**) Comparison between DEGs and DAS genes using only the fraction of regulated genes. (**C**,**D**) Gene ontology (GO) analysis of enriched biological processes of DEGs (**C**) and the DEGs vs. DAS genes intersection (**D**).

Across all the selected studies, we identified transcripts from 28,094 genes, of which roughly half were DEGs (Figure 1A). Among the DEGs fraction, 19.2% (2742 genes) were differentially regulated by AS. We also identified 2893 genes that were exclusively regulated by alternative splicing (DAS) and did not present overall transcript accumulation differences (Figure 1A). Generally, up to 50.9% of the transcriptome can be differentially expressed in response to salinity, and 19.1% of it can be regulated by AS. When focusing on the regulated portion of genes, either by transcript accumulation or AS, we noticed that the overlap between these two is only 15.9%. In comparison, the fraction of genes that are only regulated by AS is roughly the same size (16.8%) (Figure 1B). This suggests that AS regulates equally big sets of genes independently or in coordination with other regulation mechanisms.

Furthermore, we wanted to check the actual proportions of the transcriptome that were regulated by either AS or by transcript accumulation in each experiment, as opposed to looking at the universe (union) of DAS genes or DEGs from all datasets. The variation in experimental conditions and data availability (Table 1) was such that the intersection of DAS events or DEGs between datasets was very small (Figure S1B and C, respectively), and the number of DAS events was variable (Table S1). However, we were able to assess that, on average, AS alone regulates 0.79% of the transcriptome (Table S2). Contrastingly, all other mechanisms that determine differences in transcript accumulation contribute, on average, to 5.71% of the transcriptome regulation. When coordinated (AS and DEGs), they contribute to regulating 0.11% of expressed genes.

Inevitably, we asked what biologically relevant processes are enriched in these sets of genes, for which we performed a gene ontology (GO) enrichment analysis. Interestingly, among the genes that are solely DEGs, the only stress-related GO terms were associated with decreased oxygen levels and response to hypoxia, while hormone-related GO terms seemed to be more abundant (Figure 1C). Whereas no stress-related GO terms were obtained from the group of genes that were only regulated by alternative splicing (Figure S2A), in the group of genes that are both DEG and DAS, stress-related GO terms were predominant. For instance, about 600 genes were found within the “response to stress” GO term, and other big sets of around 200 to 400 genes were found within “response to cold”, “response to temperature”, “response to abiotic stimulus”, “response to oxygen-containing compounds”, and “response to external stimulus”, among others (Figure 1D).

Overall, these results suggest that even though AS only contributes to a relatively small proportion of the transcriptome regulation, it acts on a different set of genes when it is working in coordination with other transcriptional regulation mechanisms, compared to when it is acting independently. In doing so, it may contribute to fine-tuning the regulation of essential genes involved in the response to salinity, either by transcript accumulation or changing mRNA sequences.

### 2.3. Characteristics of Alternative Splicing in Salinity

**Figure 2:**
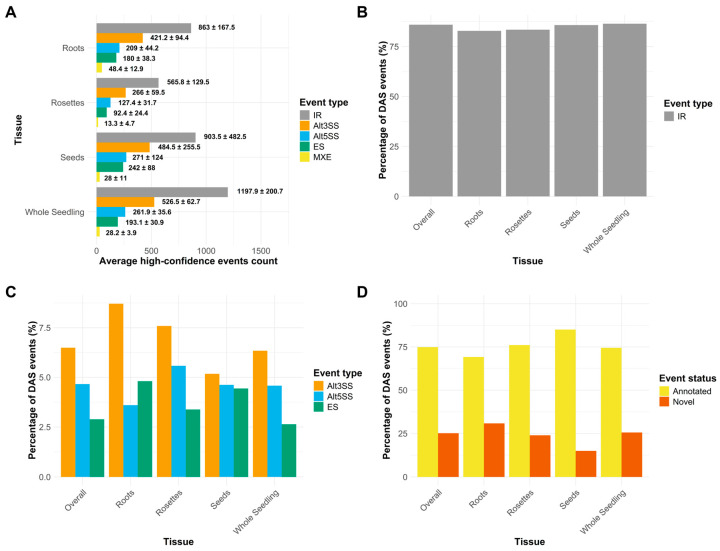
Characteristics of alternative splicing events in salinity treatments across tissues. (**A**) Average numbers of the different types of high-confidence alternative splicing events categorized by tissue. IR: Intron retention, Alt3SS: Alternative 3′ splice site, Alt5SS: Alternative 5′ splice site, ES: Exon skipping, MXE: Mutually exclusive exons. (**B**,**C**) Percentages of differential alternative splicing (DAS) events per tissue, only IR events (**B**) and the rest of events (**C**). (**D**) Percentages of novel versus annotated DAS events per tissue.

Using rMATs turbo and custom filters, we obtained the general counts of high-confidence events in each experiment, considering the events detected in all libraries and classified by event type (Table S1). With this information, we calculated the average number of events of each type by tissue. In all cases, intron retention (IR) was the most abundant event, representing between 40 and 50% (Figure 2A). The second most abundant event was an alternative 3-prime splice site (Alt3SS), followed by the alternative 5-prime splice site (Alt5SS), exon skipping (ES), and mutually exclusive exons (MXE) (Figure 2A). We then wondered whether these abundances persisted when considering the salt-responsive events, for which we looked at the ASpli output. In response to salinity, IR represents up to 80% of the total events in all tissues (Figure 2B), showing a considerable increase, whereas Alt3SS, Alt5SS, and ES maintain their relative abundances. Interestingly, when looking at individual tissues, we noticed that in the roots, ES and Alt5SS switch places, with the former being more abundant than the latter. This was not observed to be influenced by the ecotype (Figure S2A–D). We tried to perform a GO enrichment analysis on the genes on which these ES root events occurred. Still, we could not find any significant enrichment, and they did not seem to be involved in any common stress-related process (Figure S3A). Nonetheless, this slight change in event proportions could indicate how AS type selection could contribute to tissue-specific regulation.

Moreover, we investigated whether the salt-responsive events were already annotated or were “novel”, as marked by the software ASpli. For this, we compared the annotated versus novel percentages using Araport11 and atRTD2 as reference annotations. Either overall or by tissue, the atRTD2 annotation outperformed Araport11 in annotated events (Figure S3B), so we decided to stick to atRTD2 for the rest of the analysis. Considering all datasets, around 75% of DAS events were already annotated, with another 25% marked as novel (Figure 2D). Intriguingly, roots showed a slightly higher percentage of novels with over 30% of new events. These novel events were strongly associated with many salt and abiotic stress-related GO terms (Figure S3C), possibly indicating that AS regulation could contribute to salt responses in a tissue-specific manner.

### 2.4. Determinants of AS Events Detection and Generation

Considering the variability in experimental and technical conditions (Table 1 and Table S1), we first checked the size of the intersection between DAS events of the different comparisons When looking at the top ten largest datasets in terms of events, we found that the biggest possible intersection was of 174 DAS events between only two datasets (Figure S1B). No intersection was found between these ten or all 52 datasets. Similarly, DEGs followed the same tendency, with only 175 common genes, again between only two datasets (Figure S1C).

**Figure 3:**
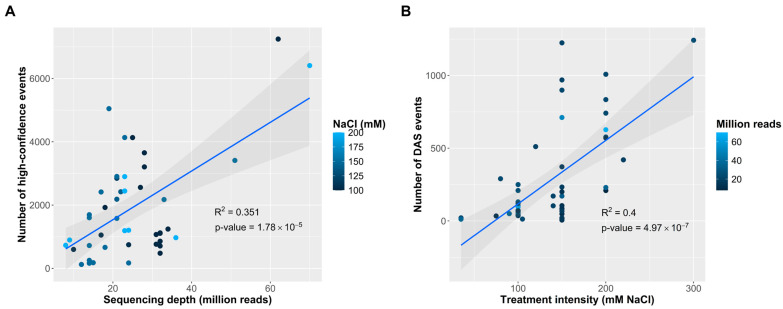
Experimental and biological parameters influencing alternative splicing events detection and production in response to salinity. (**A**) Number of high-confidence events per dataset as a function of the sequencing depth. (**B**) Number of differential alternative splicing (DAS) events per dataset versus salt treatment intensity. In both figures, we show the results of linear regression analysis and Pearson correlation coefficients (R^2^) calculated using FDR < 0.05 as threshold.

We then wondered what technical and biological factors could affect the detection and numbers of DAS events, for which we performed several correlation analyses. Considering the first technical limitation could be related to the number of RNA-seq reads that fall in specific junctions, we performed a linear correlation analysis between the sequencing depth of the RNA-seq experiments and the numbers of high confidence events. As a result, we found a positive correlation between these two variables (Figure 3A), explaining up to 35% of the variation in the events numbers—the more million reads, the higher the number of AS events that can be detected. We also performed a rarefaction curve (Figure S4A) which showed that beyond a relative threshold of 30 million average reads per experiment, the discovery of new DAS events starts ceasing and that this dynamic is affected by treatment intensity.

Moreover, treatment intensity was shown to be the more important biological and experimental parameter, as it positively correlated with the number of salt-responsive events, explaining up to 40% of the variance (Figure 3B). This tendency was also present when correlating with the numbers of DEGs (Figure S4B), indicating that both transcript accumulation and AS regulation respond to salt-treatment. We also checked correlations with the duration of treatment and plant age at treatment. Treatment duration and plant age at treatment showed a slight negative and no correlation with the numbers of DAS, respectively (Figure S4C,D). Furthermore, we performed a principal component analysis (Figure S5) to determine which of these factors could influence the specific profile of DAS regulated genes in contrast to just correlating with the total number of events or DAS events as previously. The PCA and the PERMANOVA analysis revealed that no single tested variable can explain or predict DAS profiles, possibly indicating a continuity of variations that cannot be fully captured with the available data.

Finally, considering that no single variable could explain DAS profiles and how small the intersection between datasets was, we decided to carry on further exploration working on the union of genes or events, to understand how AS can potentially act on different sets of genes grouped by tissue or abiotic stress condition. For this reason, when referring to salt or salinity “response” or “responses”, we are encompassing treatments with varying durations, intensities, and experimental conditions. This does not imply a single, universal response, but rather illustrates how DAS can potentially influence genes involved in these processes.

### 2.5. Tissue-Specific AS Regulation

**Figure 4:**
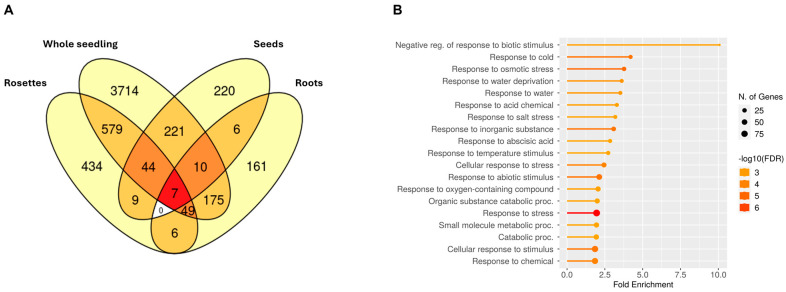
Alternative splicing regulation of genes found in different tissues. (**A**) Intersection of differential alternative splicing (DAS) genes by tissue. The more intense the color, the more datasets that participate in the intersection (**B**) Gene ontology (GO) enrichment of biological processes of DAS genes in the roots.

Given the previously described indications that AS could act differently in distinct tissues, we wondered what sets of genes were regulated in these in response to salinity. We compared the grouped DAS genes across tissues (Figure 4A) and found that most of the DAS genes identified were tissue specific. For instance, in whole seedlings, up to 77% of the DAS events were not found to be differentially spliced in roots, seeds, or rosettes.

Meanwhile, in the roots, around 39% of the DAS genes were root specific. To analyze the specific regulation in each set of DAS genes in more detail, we performed a GO enrichment analysis. As expected, the DAS genes present in two or more tissues showed several GO terms related to stress responses (Figure S6A). Unexpectedly, the DAS genes present in three or more tissues (only 110 genes) showed GO terms related to lipids metabolism and splicing, while only one GO term was related to stress (“Negative reg. of responses to water deprivation”) (Figure S6B). The DAS genes in rosettes, whole seedlings, and seeds belonged to very few stress-related GO terms (Figure S7). Contrastingly, the roots of DAS genes were strongly associated with both salt stress and abiotic stress-related biological processes (Figure 4B), such as “Responses to salt stress”, “Response to osmotic stress”, “Response to temperature stimulus”, “Response to cold”, “Response to water deprivation”, “Response to oxygen-containing compounds”, and “Response to abscisic acid”.

### 2.6. Cross-Stress Alternative Splicing Regulation in the Roots

**Figure 5:**
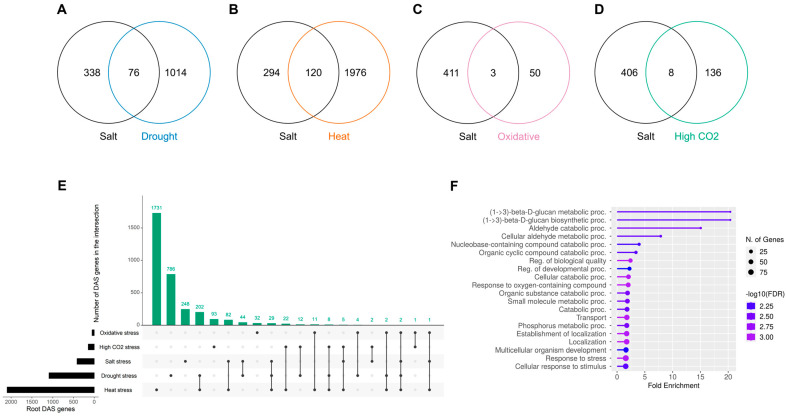
Alternative splicing regulation under different abiotic conditions in the roots. (**A**–**D**) Comparison of salt roots differential alternative splicing (DAS) genes versus drought (**A**), heat (**B**), oxidative (**C**), and high CO_2_ (**D**) DAS genes. (**E**) Overall comparison between all different abiotic conditions. (**F**) Gene ontology (GO) enrichment analysis of biological processes of DAS genes found in two or more conditions.

**Figure 6:**
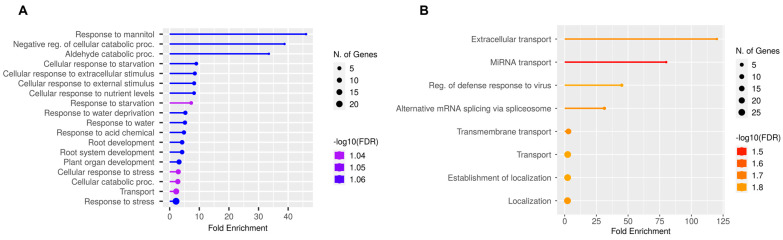
Gene ontology (GO) enrichment of differential alternative splicing genes (DAS) shared between different abiotic conditions. (**A**) Biological processes enriched in the shared DAS genes between salt and drought. (**B**) Biological processes enriched in the shared DAS genes between salt and heat.

Considering that most of the significant root-specific DAS genes that changed in response to salinity were related to stress responses and ABA, we speculated that there might be a common abiotic AS stress response. To test this hypothesis, we searched and selected datasets of roots under different abiotic stress responses such as drought, heat, oxidative, and high CO_2_ (Table S3). From a total of 414 genes undergoing DAS in response to salinity in roots, only drought and heat stress showed a relevant number of common DAS genes with salt. In the case of drought and salt, 18% of salt DAS genes are also regulated under drought (Figure 5A), while under heat stress, almost 29% of the DAS genes in response to salinity were also regulated under heat stress (Figure 5B). For oxidative stress and high CO_2_ it was only 0.7% and 1.9% of the genes, respectively (Figure 5C,D). When further analyzed, the overall overlap between stresses was mainly dominated by salt, drought, and heat (Figure 5E), with a total of 428 genes undergoing DAS under two or more stresses. Interestingly, the GO enrichment of this group of genes still showed a response to stress and cellular response to stimulus (Figure 5F). However, when we analyzed the GO enrichment of the 76 genes of salt-drought overlap, most of the GO terms were related to either stress or root development (Figure 6A). The 120 genes of the salt-heat overlap only showed GO terms related to transport and alternative splicing (Figure 6B). Ultimately, we looked at the specific overlap between salt, heat, and drought stress (Figure S8A), finding this set of genes enriched in cellular component GO terms associated with different cell–cell junctions (Figure S8B) and according to KEGG analysis, enriched in galactose metabolism genes involved in steps to produce galactose, glucose, and raffinose (Figure S8C).

## 3. Discussion

In the last decade, there has been increasing evidence about the involvement of AS in different essential plant processes, from development [31] and reproduction [32] to abiotic [12,33] and biotic stress [34] responses. Yet, its specific contribution to salinity responses remains underexplored and limited by single-experiment approaches. In this study, we leveraged all publicly available RNA-seq datasets to systematically assess AS regulation in Arabidopsis thaliana, offering a broader and more robust perspective of its participation in salinity responses. By integrating data across multiple studies, we captured the conserved and variable aspects of AS regulation under salinity conditions and its interplay with transcriptional regulation. Below, we discuss how AS may contribute to salinity responses and tissue-specific regulation, how it correlates with experimental and biological factors, and its potential role in other abiotic stress responses in the roots.

### 3.1. Alternative Splicing as a Regulatory Layer in Salinity

A prevalent question in plant transcriptomics is to what extent the different regulatory mechanisms work in coordination or separately to fine-tune the response to physiological and environmental cues [12]. This question does not escape AS in the context of salinity. Here, we showed that AS regulates sets of genes that present transcript accumulation differences in the same proportion, as it regulates genes that do not (see Figure 1). The latter fraction would usually escape detection in microarray-based studies. This follows what has been reported in single-experiment approaches [18] and in other abiotic stresses [35,36].

When comparing alternative splicing (AS) with differentially expressed genes (DEGs), we are contrasting a specific co-transcriptional mechanism with a broad spectrum of regulatory events that influence overall transcript accumulation. These include transcriptional and epigenetic modifications that affect promoter activity and accessibility, co-transcriptional processes such as differential polymerase processivity, alternative transcription start sites, transcriptional interference, mRNA editing, alternative polyadenylation, as well as post-transcriptional mechanisms such as mRNA stabilization, export, and quality control mechanisms [37,38]. Our approach, however, shows that even though its contribution in particular experimental conditions can appear relatively small, AS can potentially regulate up to 20% of the detected transcriptome in response to salt, and it contributes to co-regulating around 10% of the genes that show differential transcript accumulation.

The nature of AS regulation is complex and dependent on the type of modification introduced, it can act in the form of transcript degradation via nonsense-mediated decay (NMD) or nuclear retention [39], changes in transcript stability, subcellular localization and translation efficiency [40], and the production of truncated or functionally distinct protein isoforms [41,42,43]. Another key finding that we reported was that AS regulates genes directly related to salinity and stress responses when acting in coordination with transcript accumulation. This hints at a potential evolutionary adaptive mechanism of plants as sessile organisms which respond to environmental conditions by diversifying their transcriptome and proteome. An increasing body of evidence supports this idea, with several key splicing factors and other downstream signaling regulators suffering salt induced AS events that have an impact on salt tolerance. For instance, a serine/arginine-rich (SR)-like protein, SR45, that acts as a splicing factor in *Arabidopsis* produces two isoforms via AS in response to salt of which only one can mediate salt tolerance [43]. A RING-Type E3 ligase, called salt-responsive alternatively spliced gene 1 (SRAS1), produces two coding isoforms with opposing activities. SRAS1.1 overexpression can make plants more salt-tolerant via targeting CSN5A (a spliceosome component), whereas SRAS1.2 overexpression made them more sensitive [44]. Cases of AS mediated salt-tolerance in other species have also been reported. For example, a plasma membrane H+-ATPase (PMA) from *Vitis vinifera* produced two splicing variants detected in the roots under salinity (VvPMA1α and VvPMA1β), which differed in their H+-ATPase activity [45]. Similarly, two splicing variants of maize high-affinity potassium transporter (HKT) (ZmHKT1;1a and ZmHKT1;1b) show differing levels of salt tolerance in transgenic tobacco [46].

Even though, as previously mentioned, AS regulation is not only limited to coding sequences changes, our analysis aligns with the body of evidence stating that there is strong AS regulation in salt-responsive genes. We therefore highlight the limitations of relying solely on DEGs to study salinity responses in *A. thaliana*, as alternative splicing plays a crucial role in fine-tuning transcriptomic plasticity under salinity.

### 3.2. Mechanistic Insights into Alternative Splicing Regulation in Salinity

Understanding the landscape of alternative splicing regulation in response to salinity requires identifying the differentially spliced genes and characterizing the specific types of AS events that occur and respond to this stimulus. Our analysis shows that, on average, IR is the most abundant type of AS in *A. thaliana*, followed by Alt3SS, Alt5SS, and ES (see Figure 2). This general hierarchy of AS types has also been reported in salinity in grapevine and cotton, and in other abiotic conditions [21,22,47,48,49]. Contrastingly, other eukaryotes and even plants have different predominant events, such as ES in mammals [50]. Here, we go further to show that when focusing on salt-responsive events, there is an increase in the proportion of IR events across all tissues.

IR is of great importance in plant transcriptome regulation, as it can (1) cause nuclear retention of transcripts, (2) introduce premature termination codons (PTC) that either trigger degradation through NMD or (3) generate truncated proteins with potentially altered functions [13]. This widespread increase in IR under challenging conditions has been previously discussed to result from a stress-induced reduction in spliceosome efficiency, leading to decreased recognition of strong splice sites [12]. However, in agreement with our findings, many stress-responsive genes appear to naturally possess weaker splice sites, suggesting that AS regulation under salinity may be more targeted than previously thought [51].

Intriguingly, in *Arabidopsis,* the coordination between AS and NMD through introduced PTC has been shown to be functionally important for pathogen defense. As reviewed previously, in normal conditions, NMD depletes salicylic acid (SA) synthesis genes and other defense genes. Then, upon biotic stimuli, NMD is repressed, and the pathogen response is activated [52]. We hypothesize that this widespread IR increase in response to salt and other abiotic stresses could also be of importance for tolerance through a similar coordination mechanism (AS->IR->PTC->NMD), in which AS produces IR-introduced PTC that then target specific splicing variants for degradation, prioritizing the production of more context-specific relevant isoforms. Nonetheless, further research will be needed to more deeply address the potential consequences of these overall increases in IR events in plant salt and abiotic stress responses.

In the roots, we observed a switch in the relative abundance of ES events and Alt5SS in salinity conditions. Even though we did not find any significant association between those ES-affected genes and a particular biological process, this pattern raises questions about the functional role of ES in salinity, particularly in the roots. Comparably, other plant species such as tomato, date palm, and wheat have ES as the predominant event in response to salt treatments [20,53,54]. Alt3SS, Alt5SS, and ES are generally associated with proteomic diversification, since they directly affect protein sequences more frequently than IR [55]. However, they have also been proposed to function redundantly with IR in modulating protein levels by regulating transcript degradation [51]. Nonetheless, the observed tissue-specific shift in AS event proportions suggests a more refined layer of AS regulation. Not only do different genes undergo AS in different tissues, but the AS event type hierarchy may be context dependent.

Another important insight from our analysis is the variable number of the novel (non-annotated) salt-responsive AS events across tissues, with roots showing the highest proportion of these events (see Figure 2D). Notably, these novel events tend to occur in stress-associated genes (see Figure S3C), reinforcing the idea that some AS events may be stress-induced and condition-specific rather than constitutively present. One possible explanation for this could be that roots serve as the primary interface with the salt affected soil environment, making them the first organ to sense and respond. Moreover, unlike shoots, which can modulate responses by fine-tuning stomatal aperture and water transport, roots remain embedded in the saline soil and must therefore be better prepared to mitigate toxicity, osmotic and oxidative changes. Consequently, roots may have developed a more dynamic and stress-responsive transcriptome, including alternative splicing patterns that are induced specifically under salt stress. Additionally, we found that the number of detected novel AS events varies depending on the reference annotation used (see Figure S3B), highlighting potential gaps in current *Arabidopsis* transcript annotations, typically based on non-stressed conditions. Finally, while some of these differences may reflect biases introduced by dataset selection or experimental conditions, they also suggest that a subset of salt-induced AS events remains underrepresented in existing genome annotations.

### 3.3. Experimental and Biological Considerations in Alternative Splicing Studies

The study of AS events using RNA-seq data is inherently influenced by technical and experimental factors that can influence how many events are produced and if we can detect them. Our analysis found that sequencing depth (read number) and salt treatment intensity were the two most relevant parameters affecting AS detection (see Figure 3).

In most short-read-based methods for AS quantification, like the ones we use here, the identification of events depends on how likely it is for a particular junction to have reads that align with it and allow for accurate quantitative measurements. It has been previously described that read coverage is essential in increasing the likelihood of identifying splicing variants, especially when they are low in abundance [56,57]. Our findings support this, as we observed a positive correlation between the number of sequencing reads and the number of detected DEGs and AS events (see Figure 3), suggesting that technical limitations could cause the underrepresentation of AS numbers and diversity in lower-depth datasets, and that this is a defining factor to consider when designing an RNA-seq experiment for AS detection. Based solely on the tendencies observed in our rarefaction curve (see Figure S4A), we would cautiously recommend considering a minimum average of 30 million reads per Illumina sequencing library to have a better chance at achieving the most variation in salinity treatments in *Arabidopsis*. This is in line with recommendations made by the ENCONDE Consortium (Stanford, CA, USA) [58] and Illumina Inc. (San Diego, CA, USA) [59] in order to obtain a global in-depth overview of transcriptome changes. However, it is important to acknowledge that other parameters may also influence the number of DAS events and genes.

On the other hand, salt treatment intensity was the main experimental factor influencing AS detection, mirroring previous findings regarding DEGs (also see Figure S4B), where the more potent the challenging or stress conditions, the more pronounced transcript accumulation changes [60]. Our results revealed that higher salt concentrations can result in more DAS events, whereas low-intensity treatments have a milder effect. This is to be expected, as mild salt exposure may not impose sufficient physiological stress to trigger a more extensive transcriptional response. Interestingly, neither plant age at treatment nor treatment duration significantly influenced the number of generated DAS events (see Figure S4C,D), possibly indicating that at least the numerical dynamic of AS events is mostly independent of these factors.

While sequencing depth and treatment intensity were the most relevant parameters affecting detection, we showed that no single experimental variable can influence the variability of DAS profiles (the specific genes affected rather than the total number of events or genes). The two first principal components from our PCA (see Figure S5) could only explain around 10% of the variability each, suggesting that plant age at treatment, treatment duration, and salt intensity do not individually dictate how AS targets certain genes. This is not to say that these variables do not affect or impact AS regulation, considering that they do have an impact from a biological point of view. We think this may reflect the continuous nature of splicing variation, which we cannot capture through discontinuous sampling, given the inherent constraints of working with publicly available transcriptomic data. In any case, our goal here is to establish a broader overview of genes that are regulated by AS when exposed to salinity in any given condition.

### 3.4. Tissue-Dependent Nature of Alternative Splicing Regulation in Salinity

Most RNA-seq-based transcriptomics studies have focused on sequencing whole seedling samples, with individual tissues such as roots, rosettes, seeds, and others being underrepresented or nonexistent in public databases. By compiling and analyzing all available public salt treatment datasets spanning multiple tissues, we showed that even though whole seedlings can provide a global overview of transcriptional and AS regulation, it does not capture more fine or precise changes occurring at the level of individual tissues or even cell-types (see Figure 4A). This is a prevalent problem with bulk RNA-seq approaches. Biases of at least three types are inevitably introduced: (1) prevalence of the transcriptional program of the most abundant tissue or cell-types, (2) prevalence of the most transcriptionally active elements, and (3) prevalence of the transcripts and AS events that are more abundant; all of which can change over time and across conditions. Nevertheless, using our approach, AS events captured across heterogeneous conditions remain highly relevant, because we provide robust core datasets that average out physiological variation due to experimental and tissue differences, offering insights into AS events that are consistently involved in the plant’s response to salinity across diverse conditions.

A good portion of DAS genes are shared between whole seedlings and rosettes or whole seedlings and roots, however, a relevant proportion of these genes are only captured in a tissue-specific manner. This goes in line with other reports that emphasize the tissue-specific [51,61] and even cell-type specific [62] nature of many alternative splicing events, also with events being generated in a developmental stage-specific manner [31]. We hypothesize that distribution bias could be introduced when using whole seedling biomass, especially in older plants, favoring shoot structures such as hypocotyls, cotyledons, emerging leaves or rosettes and skewing sequencing towards the most abundant tissues and events.

Aside from looking at the tissue-specific aspect of AS regulation, we also showed that there seems to be a core set of DAS events that is shared between at least three or more tissues. This core salinity set of genes seems to be involved mainly in two biological processes: splicing regulation and lipid metabolism. This self-regulation of splicing machinery through alternative splicing has previously been extensively described [18,63]. There are characterized examples of how upstream splicing regulators such as splicing factors can suffer isoform switches through AS and mediate salt stress responses and adaptation [43]. Interestingly, lipid metabolism is considered an essential factor in the adaptation response to salinity [64], with significant lipidome and membrane lipid composition changes linked to enhanced or altered salt tolerance in other species [65]. Our findings suggest that AS may contribute to lipidome remodeling under salinity, adding a new dimension to this overlooked adaptive mechanism.

### 3.5. Shared and Unique Alternative Splicing Regulated Genes Across Stress Conditions

Consistent with the role of the roots as the first tissue to sense salinity in the soil and trigger responses in the plant, we showed that a significant proportion of DAS events found in the roots are involved in stress responses (see Figure 4B). Also, this response was specific to the roots, reinforcing the notion of AS contributing to salinity responses in a tissue-specific manner.

Physiologically, salt stress can be broadly divided into three sub-components, first, an osmotic imbalance caused by decreased soil water potential that triggers roots to shoot ABA-mediated stomatal closure, affecting transpiration and photosynthesis rates and triggering the transport and synthesis of osmolytes. The second is the ionic component caused especially by the accumulation of sodium ions, depolarizing the membrane and disrupting potassium homeostasis, consequently triggering the salt overly sensitive (SOS) response to counteract ionic imbalance and toxicity. And thirdly, an oxidative stress component by overproduction of reactive oxygen species such as superoxide radicals and hydrogen peroxide that can damage proteins and nucleic acids, and must therefore be avoided through the activation and expression of detoxification enzymes and antioxidants [66,67]. Accordingly, we found the expected responses to the osmotic, ionic, and oxidative aspects of salt stress GO terms within the DAS regulated root genes.

Given the response to abiotic stress and response to ABA GO terms, we wondered how salt-specific this AS regulation was. Comparison across other stress conditions showed that there is a core AS-regulated response involving two or more stresses, which shows most genes are involved in not only stress response, such as the ones under response to stress and oxygen-containing compounds GO terms, but also in the regulation of development, transport, and other metabolic pathways (see Figure 5). These developmental and transport-associated GO terms could suggest that AS is not only involved in the immediate stress response, but also contributes to root development and nutrient uptake adjustments necessary for stress adaptation [68,69].

When comparing the different intersections, we show that salt and drought AS regulation share a subset of around 18% of the DAS genes, mostly related to root development and osmotic and water stress processes (see Figure 5A and Figure 6A). Since both stimuli can induce osmotic imbalance [70,71], it could suggest a possibly conserved AS regulation mechanism to mediate osmotic resistance. On the other hand, even though salt shared more DAS genes with heat treatment, around 29%, the shared component was functionally distinct, enriched in GO terms related to localization and transport (see Figure 5B and Figure 6B). This suggests that AS could be more involved in regulating molecular and cellular transport to coordinate resource allocation for stress mediation, mobilization of osmoprotectant molecules, or even ion transport [72,73].

Finally, the shared DAS response across heat, drought, and salinity was enriched in genes related to cell junctions and plasmodesmata, as well as galactose metabolism. Regulation of intercellular connectivity and transport may reflect a broader strategy to modulate resource flow and stress signaling, while shifts in galactose and galactose derived molecules such as raffinose could align with osmoprotection, ROS scavenging, and membrane remodeling under abiotic stress [74]. However, despite this overlap with heat and drought treatments, most DAS genes remain stress-specific, indicating that AS regulation in the roots remains mostly condition specific.

## 4. Materials and Methods

### 4.1. Data Retrieval

We conducted a comprehensive search of public transcriptomic data available in the National Center for Biotechnology Information (NCBI), specifically in the Sequence Read Archive (SRA) database, BioProject database, and the Gene Expression Omnibus (GEO). We also checked other repositories such as the European Nucleotide Archive, the China National Gene Bank (CNGB), and ArrayExpress. Custom filters were applied to select only transcriptomic data generated through RNA-seq experiments from short-read technologies using different *Arabidopsis thaliana* tissues. The keywords “arabidopsis”, “salt”, “salt stress”, “salinity”, “NaCl”, “sodium chloride”, and others, were used in the case of salt stress-related datasets. Moreover, for the comparison between salt and other abiotic stresses using only root tissue, we used related keywords such as “root”, “drought stress”, “dehydration”, “heat”, “high temperature”, “oxidative”, “carbon dioxide”, and others.

In each case, search results were manually checked to ensure the data fulfilled the previously mentioned criteria and others, such as having at least two biological replicates per comparison and valid control versus treatment experimental setting in the wild-type genotype background. After this selection, we carefully collected all relevant biological, technical, and experimental metadata of the surviving datasets/Bioprojects (Table S1).

### 4.2. Pre-Processing and Differential Expression Analysis

The sequencing data were downloaded using the SRA toolkit NCBI, v. 3.0.0 and were pre-processed as previously described by Contreras-Lopez et al. [75]. Accordingly, the sequencing reads were quality-checked and trimmed to remove low-quality and adapter sequences using FastQC v. 0.12.1 [76] and TrimGalore v. 0.6.10. [77], respectively. Clean reads were mapped to the TAIR10 reference *Arabidopsis* genome using HISAT2 v. 2.2.1. [78], after which gene counts were generated with Rsubread v. 2.14.2. [79], using AtRTD2 [80] as reference annotation. To account for possible ecotype differences, we checked that mapping and feature assignment percentages were consistent. Differentially expressed genes (DEGs) were then obtained with DESeq2 [81] by comparing between control and treatment. Filtering was then carried out using a fold change in Log2FC > 1.5 to match that of the splicing detection tools (for fair comparison), and an adjusted false discovery rate (FDR) < 0.01.

### 4.3. Detection of Alternative Splicing and Differential Alternative Splicing

Alternative splicing detection was carried out using rMATS turbo v4.3.0 [82] with custom parameters individually adjusted to each dataset. Specifically, -t (single or paired)--libType (fr-unstranded, fr-firststrand or fr-secondstrand, determined using RSeQC [83])--readLength (according to each library)--variable-read-length--allow-clipping and with--novelSS for the detection of novel events, and using both AtRTD2 and Araport11 annotations for comparison purposes. After detection, we filtered the lists to obtain high-confidence events, considered as those found in genes with read counts ≥ 10 across all samples and with percent spliced in (PSI) values between 0.05 and 0.95, as recommended by the developers.

Furthermore, differential alternative splicing (DAS) analysis was carried out using the ASpli v2.6.0 [84] R-Bioconductor library with custom parameters for each dataset regarding minReadLength, libType, strandMode, and maxISize = 50,000 in the gbcounts and jCounts functions. Then, the integrateSignals function was used to obtain DAS events using default thresholds for coverage, false discovery rate, differential splice site usage, and differential intron retention. Possible ecotype-associated differences were checked by testing differences in DAS numbers and event type proportions, prior to further processing and data integration.

### 4.4. Data Exploration, Parameters Evaluation, and Data Integration

Exploration and data analysis was carried out in R version 4.3.1, primarily using the dplyr package [85]. Visualization was carried out using the packages ggplot2 [86]. Scatter plots, bar plots, and linear regression models were used to examine trends in sequencing depth, treatment intensity, treatment duration, and plant age at treatment in relation to either DAS events numbers or high-confidence events numbers. Pearson correlation coefficients were calculated for numerical variables, and significance was determined with an FDR threshold of 0.05. To more thoroughly understand the impact of sequencing depth in the discovery of DAS events, we generated a rarefaction curve using the cumulative number of unique (not previously detected) DAS events as a function of average million reads per library, and a locally estimated scatterplot smoothing line was used to observe tendencies.

Aside from looking at the correlation between biological and experimental variables in the numbers of DAS genes and events, we performed a principal component analysis (PCA) to visualize data aggrupation using their splicing profile (Log2FC of DAS genes per dataset). To statistically test what variables significantly explained DAS profiles, we performed a permutation multivariate analysis of variance (PERMANOVA) using Bray–Curtis dissimilarity to statistically test what variables significantly explained DAS profiles, with 999 permutations and a *p*-value threshold or *p* < 0.05.

Since no single variable or mix of them could explain DAS profiles, unless otherwise stated, we used the union of all unique DAS genes or DAS events, grouping by tissue or abiotic-stress condition for all functional and splicing characteristics analyses.

### 4.5. Gene Ontology (GO) Enrichment Analysis

For gene sets or events sets analyses, we used the package “ggVennDiagram” [87] for two-set comparisons and UpSetR [88] for visualization of multi-set overlaps. The “Intervene” Shiny app [89] was also used to generate intersection visualizations across data from different tissues or stresses.

Finally, Gene Ontology (GO) enrichment analysis and KEGG analysis were performed using “ShinyGO v0.77” [90], applying an FDR threshold of 0.05 to identify significant biological processes or cellular components. Enrichment results were visualized using plots generated in the ShinyGO application.

## 5. Conclusions

Our study highlights the importance of AS in salinity conditions, with independent and coordinated roles along with transcript accumulation. We showed that AS regulates a significant proportion of genes using mostly IR as the predominant event and with strong tissue specificity, especially in the roots, where most AS-regulated genes participate in stress responses. We found core sets of genes across tissues and abiotic stress conditions, with heat and drought presenting the most overlap with salinity, suggesting both a broader AS-mediated response to abiotic conditions. We also showed that experimental parameters such as sequencing depth and salt treatment intensity can influence AS detection and numbers. Future research should investigate the functional consequences of AS-driven changes, considering its tissue-specific and condition-specific nature, to provide deeper insights into how plants respond to salinity.

## Figures and Tables

**Table 1:** Summary of relevant experimental and biological parameters across the different selected RNA-seq experiments in salinity.

Condition	Number of Categories	Categories
Tissue	4	Roots, whole seedlings, rosettes, seeds
Treatment intensity	12	From 35 to 300 mM NaCl
Treatment duration	19	From 15 min to 16 days
Treatment method	5	Solidified media, hydroponic, imbibition, irrigation
Plant age at treatment	13	Pre-germination, from 4 dpg ^1^ to 4 weeks dpg

^1^ dpg: days post germination.

## Data Availability

Data is contained within the article and Supplementary Materials.

## Supplementary Materials

The following supporting information can be downloaded at: https://www.mdpi.com/article/10.3390/plants14071064/s1.
