# Supplementary material for: Exploring Alternative Splicing in Response to Salinity: A Tissue-Level Comparative Analysis Using Arabidopsis thaliana Public Transcriptomic Data
Source: Plants (Basel). 2025 Mar 30;14(7):1064. doi: 10.3390/plants14071064 (PMC11991229; doi:10.3390/plants14071064)
Supplement: Supplementary file 1 [file plants-14-01064-s001.zip › Supplementary figures.pdf]

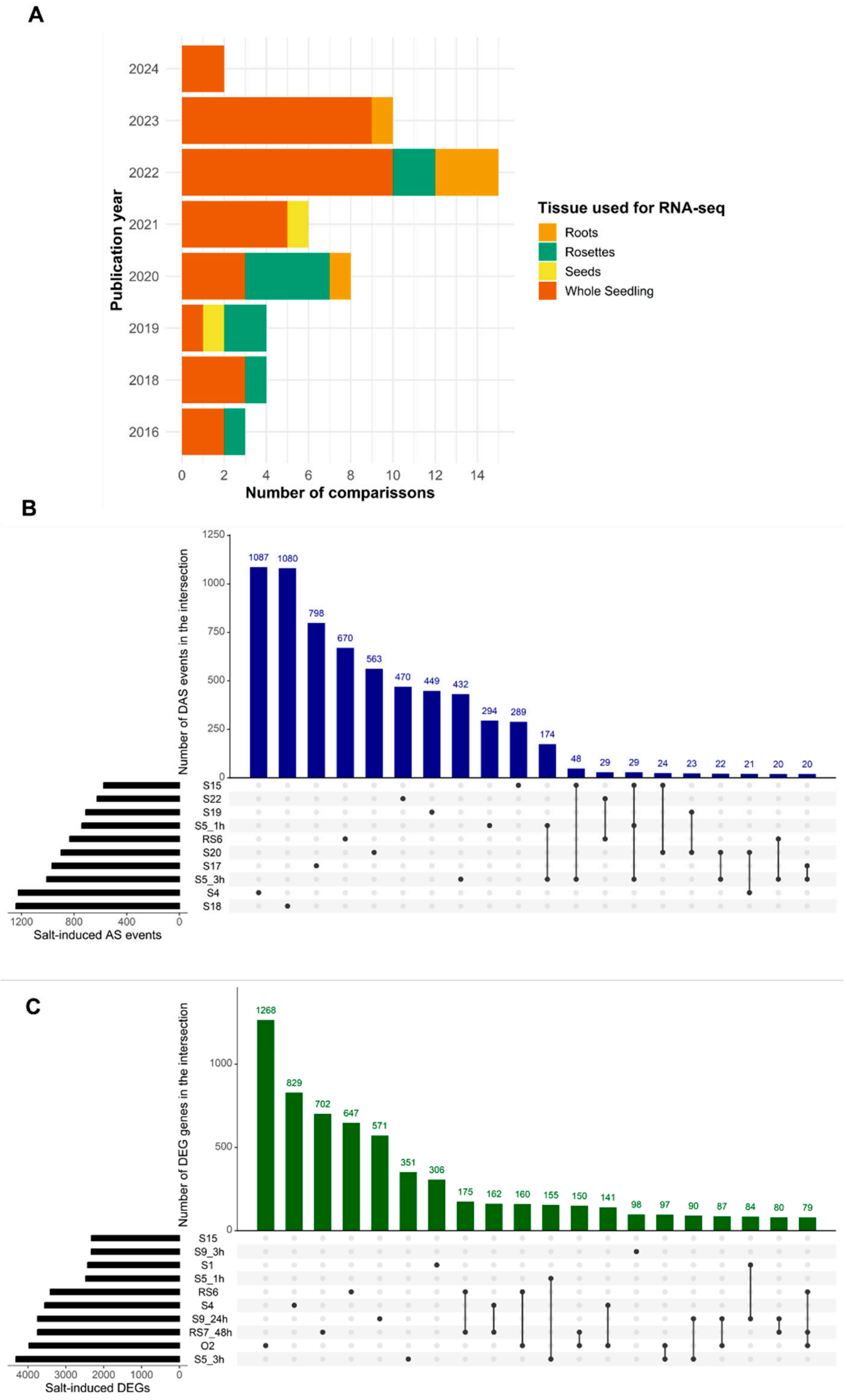

Figure S1: Summary and exploration of the selected public sequencing data. (A) Distribution of the chosen datasets categorized by tissue and year the experiment was uploaded to public databases. (B) Intersection between the top 10 largest differential alternative splicing (DAS) gene sets. (C) Intersection between the top 10 most prominent differentially expressed genes (DEGs) sets.

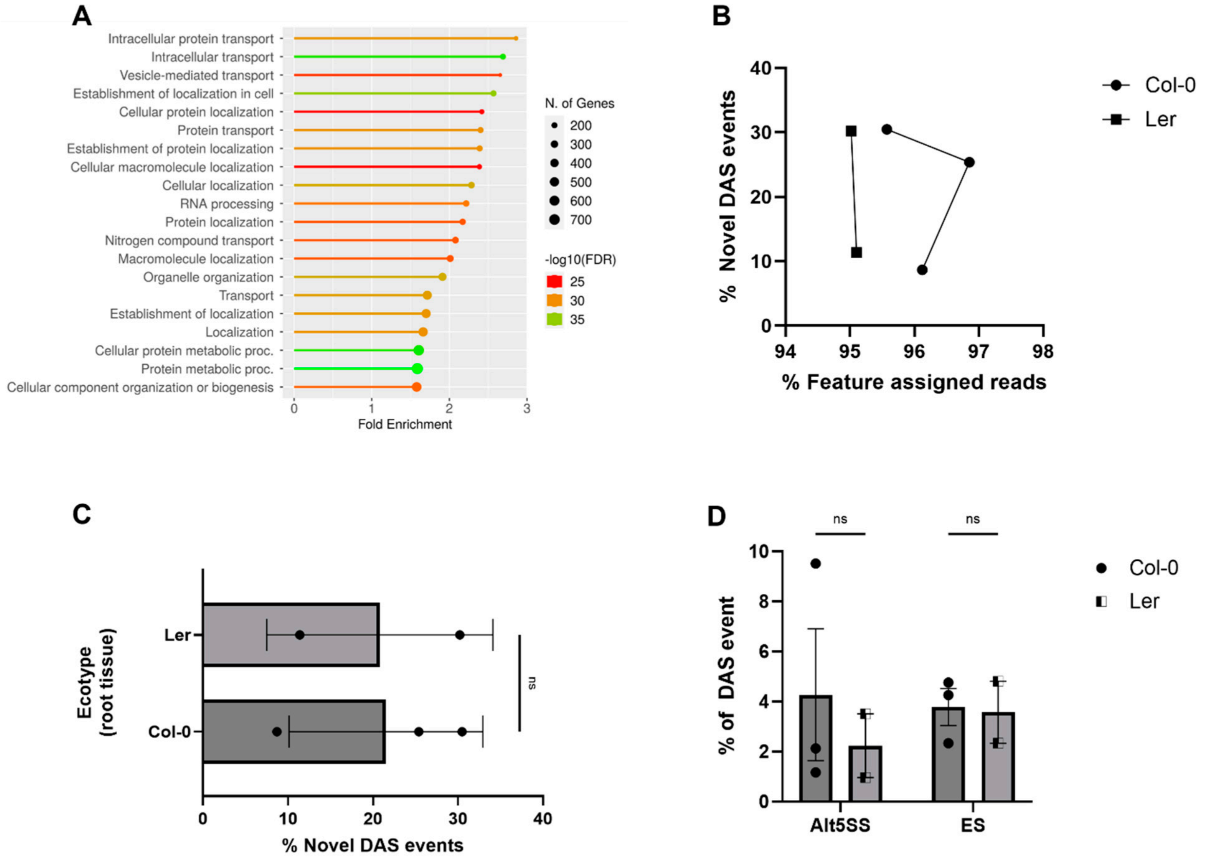

Figure S2: Gene ontology (GO) enrichment analysis of differential alternative splicing (DAS) genes and ecotype derived effects verifications. (A) Biological processes associated with the portion of DAS-only genes. (B) Number of novel events as a function of feature-assigned reads (read that map to a gene) by ecotype. (C) Percetage of novel DAS events divided by ecotype. (D) Proportion of event types as a function of ecotype.

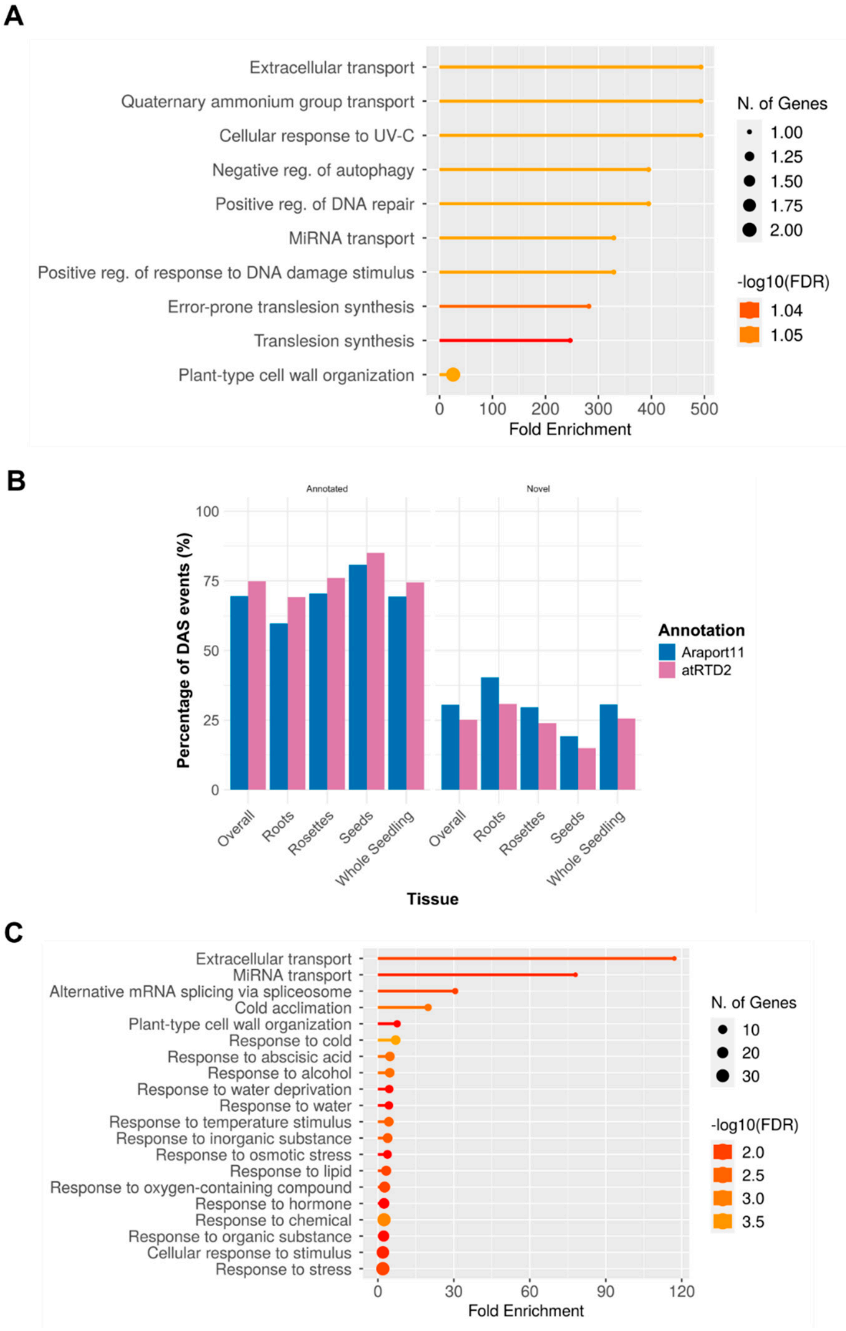

Figure S3: Functional exploration of differential alternative splicing (DAS) events and their annotation status. (A) Biological processes associated with roots exon skipping events. (B) Percentages of annotated (left panel) and novel (right panel) DAS events per tissue, using either Araport11 or atRTD2 as reference. (C) Biological processes associated with roots novel DAS events.

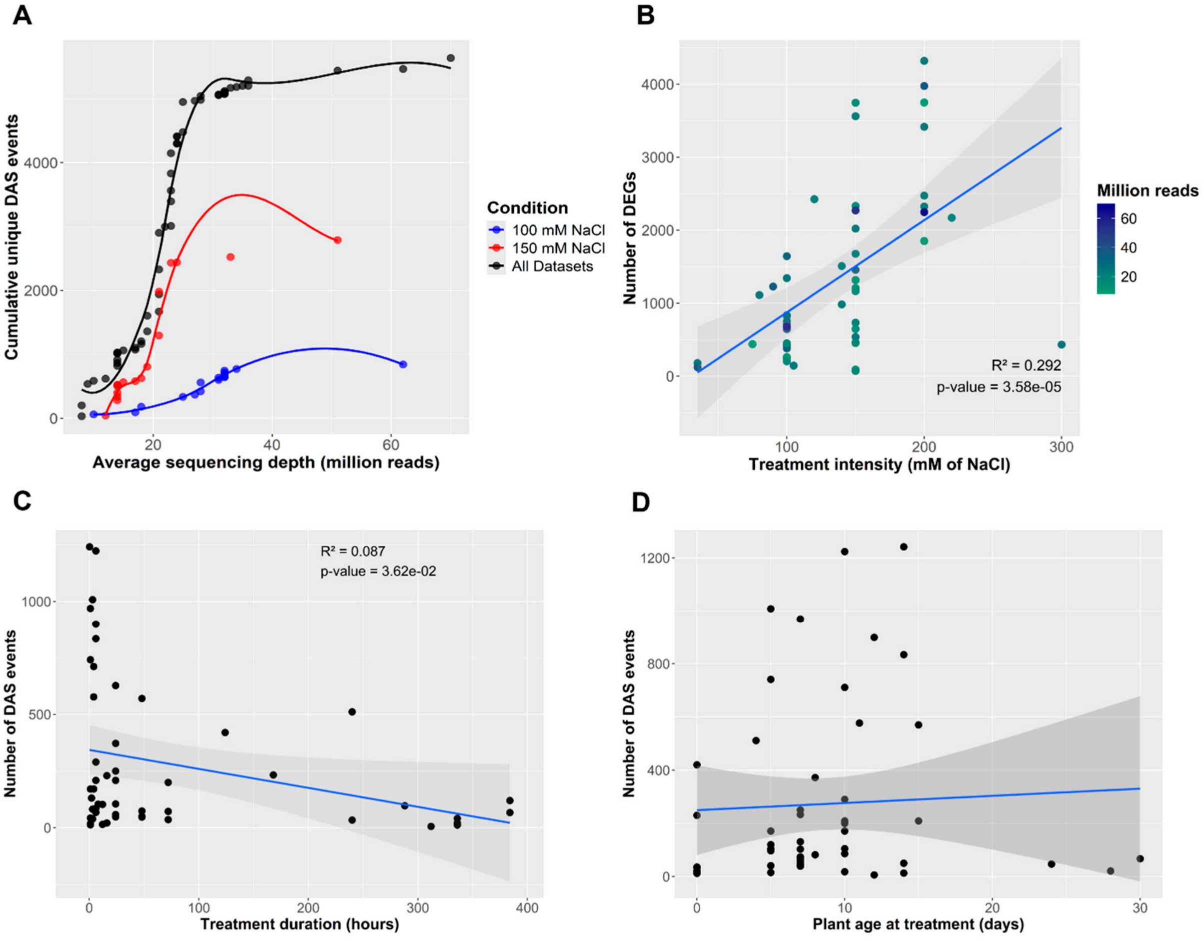

Figure S4: Exploration of transcriptional response is a function of experimental parameters. (A) Rarefaction curve of DAS events discovery as a function of sequencing depth. (B) Numbers of differentially expressed genes (DEGs) in response to treatment intensity. (C) Numbers of differential alternative splicing (DAS) genes as a function of treatment duration. (D) Numbers of DAS events versus plant age at treatment. In B and C, we show the results of linear regression analysis and Pearson correlation coefficients (R^2) calculated using FDR < 0.05 as the threshold.

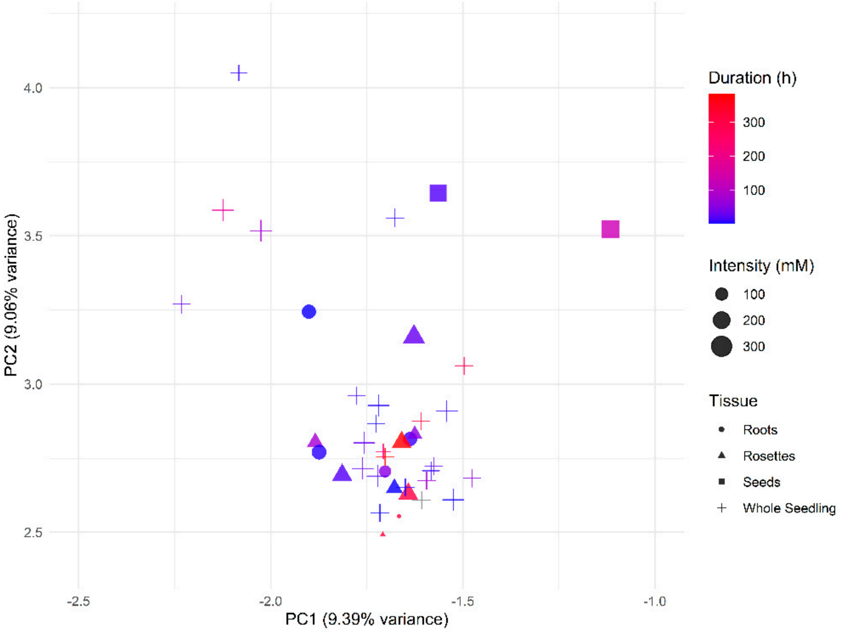

Figure S5: Evaluation of dataset similarity in terms of differential alternative splicing (DAS) genes profiles. Principal component analysis (PCA) considering the DAS profiles of each dataset, colored by treatment duration in hours, sized by treatment intensity (mM NaCl) and shaped by tissue.

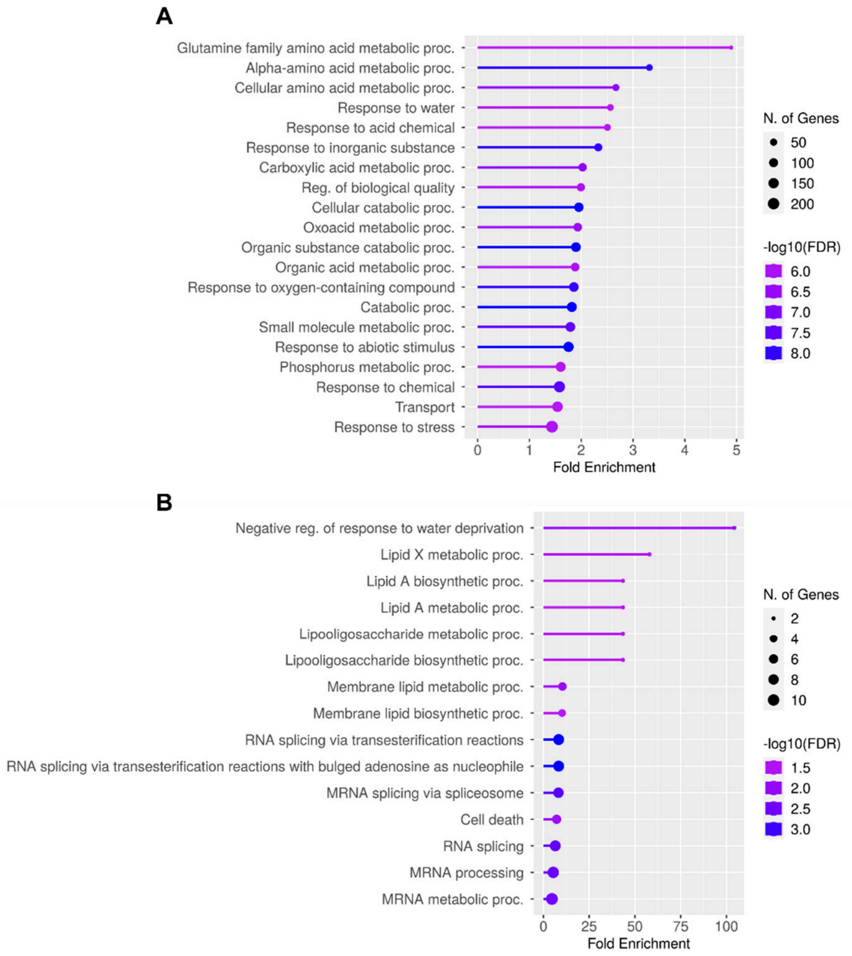

Figure S6: Functional exploration of cross-tissue differential alternative splicing (DAS) genes through gene ontology (GO) analysis. Biological processes associated with DAS genes found in two or more tissues (A) or three or more tissues (B).

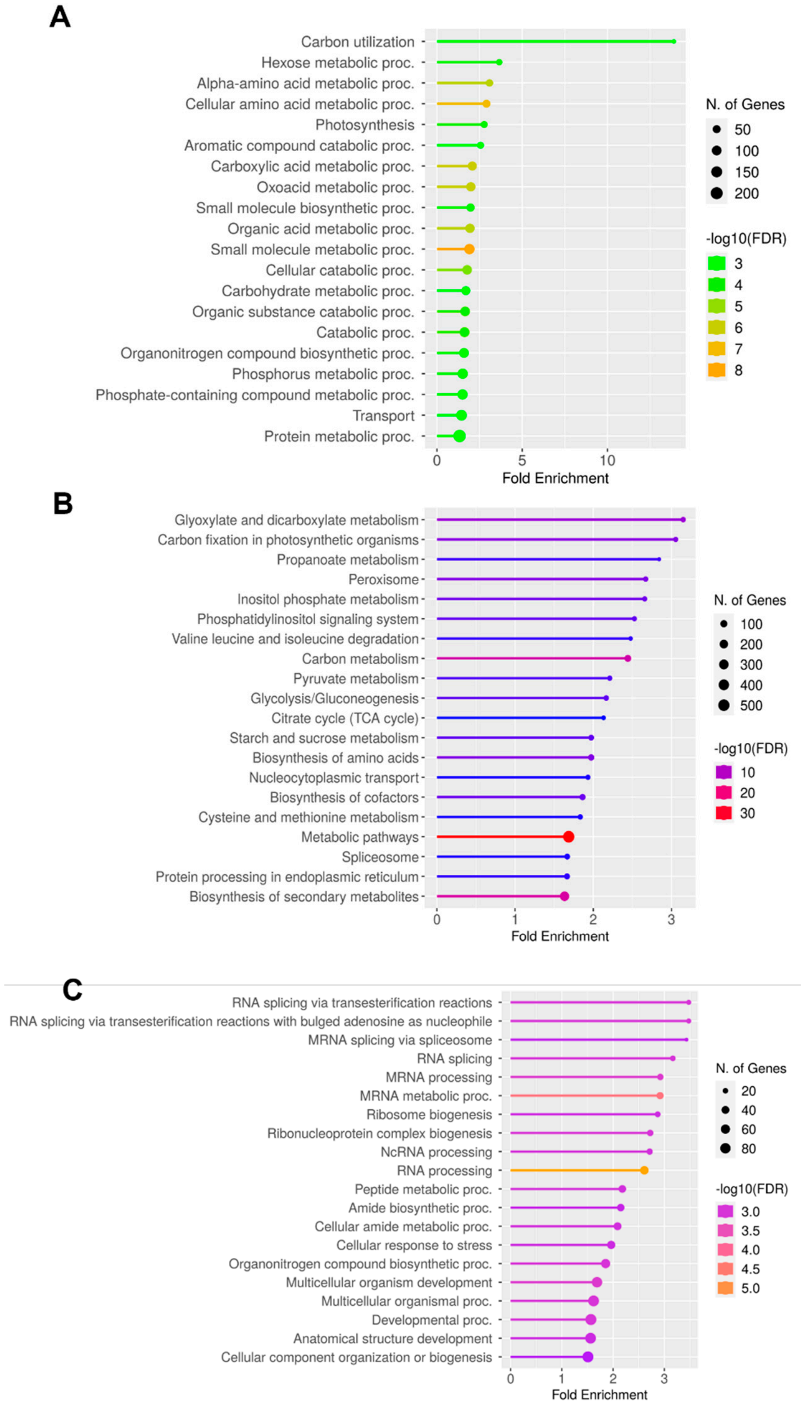

Figure S7: Functional exploration of tissue-specific differential alternative splicing (DAS) genes through gene ontology (GO) analysis. Biological processes associated with either rosettes-specific (A), whole seedling-specific (B), or seeds-specific (C) DAS genes.

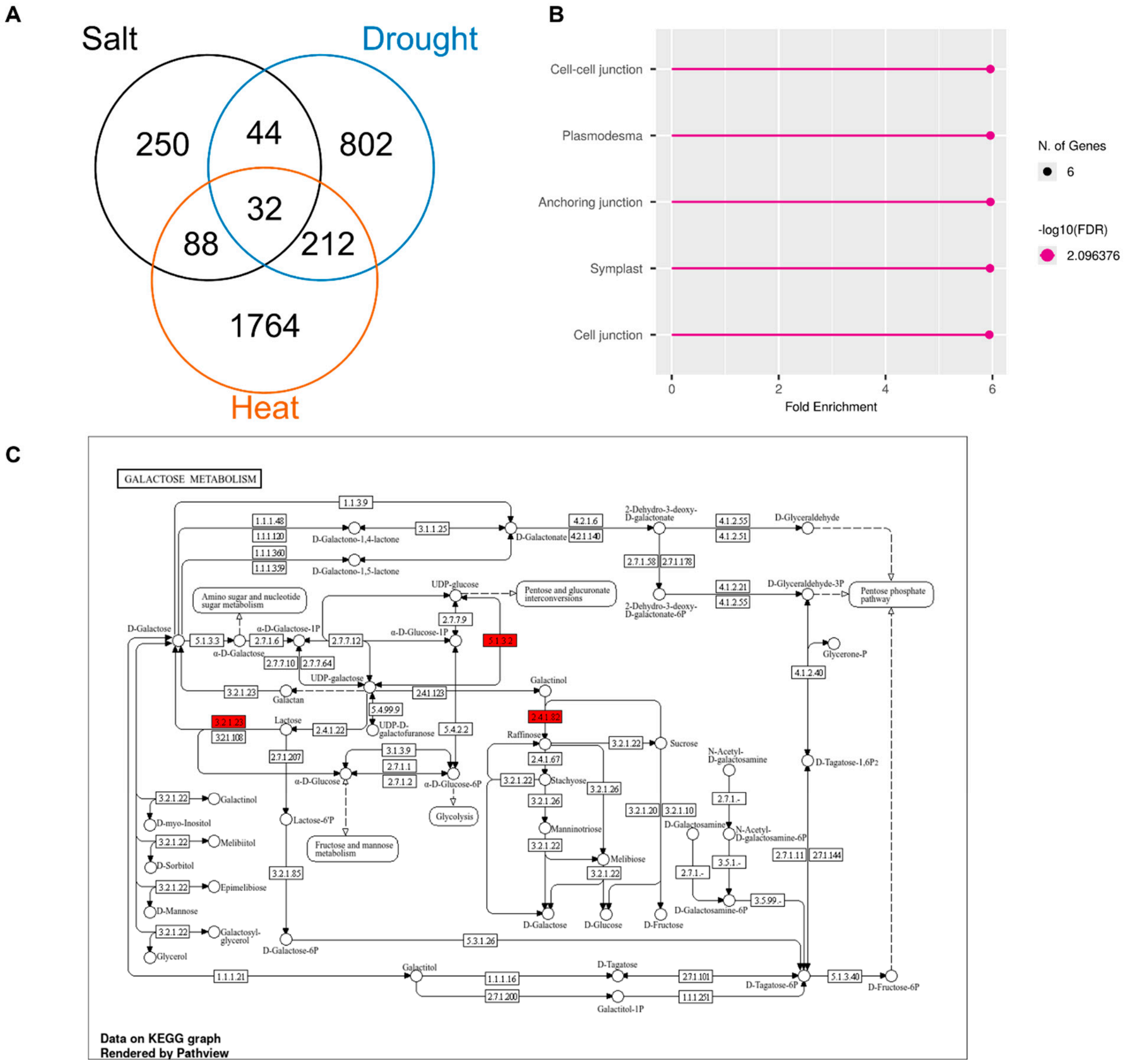

Figure S8: Functional exploration of differential alternative splicing (DAS) genes in the intersection between salt, heat and drought. (A) Venn diagram displaying the intersection between salt, heat and drought DAS genes. (B) Celullar component GO terms associated with cross salt-heat-drought intersection. (C) KEGG enriched pathway.
